# Molecular Mechanisms of Aberrant Neuroplasticity in Autism Spectrum Disorders (Review)

**DOI:** 10.17691/stm2021.13.1.10

**Published:** 2021-02-28

**Authors:** A.A. Anashkina, E.I. Erlykina

**Affiliations:** Senior Teacher, Department of Biochemistry named after G.Y. Gorodisskaya; Senior Researcher, Central Scientific Research Laboratory, Privolzhsky Research Medical University, 10/1 Minin and Pozharsky Square, Nizhny Novgorod, 603005, Russia; Professor, Head of the Department of Biochemistry named after G.Y. Gorodisskaya, Privolzhsky Research Medical University, 10/1 Minin and Pozharsky Square, Nizhny Novgorod, 603005, Russia

**Keywords:** autism spectrum disorders, aberrant neuroplasticity, glutamate excitotoxicity

## Abstract

This review presents the analysis and systematization of modern data on the molecular mechanisms of autism spectrum disorders (ASD) development. Polyetiology and the multifactorial nature of ASD have been proved. The attempt has been made to jointly review and systematize current hypotheses of ASD pathogenesis at the molecular level from the standpoint of aberrant brain plasticity. The mechanism of glutamate excitotoxicity formation, the effect of imbalance of neuroactive amino acids and their derivatives, neurotransmitters, and hormones on the ASD formation have been considered in detail. The strengths and weaknesses of the proposed hypotheses have been analyzed from the standpoint of evidence-based medicine. The conclusion has been drawn on the leading role of glutamate excitotoxicity as a biochemical mechanism of aberrant neuroplasticity accompanied by oxidative stress and mitochondrial dysfunction. The mechanism of aberrant neuroplasticity has also been traced at the critical moments of the nervous system development taking into account the influence of various factors of the internal and external environment. New approaches to searching for ASD molecular markers have been considered.

## Introduction

Autism spectrum disorders (ASD) is a comprehensive problem of modern psychology, neurology, and related sciences which is being much discussed [1]. This is connected with the nosological differentiation of ASD, lack of reliable molecular markers, diagnostic difficulties, and poorly studied etiology and pathogenesis [2–4].

In 80s of the XX century, autism was considered a relatively rare disease. In recent decades, however, the percentage of ASD diagnosing is growing steadily [5–9]. According to the current epidemiological studies, the rate of revealing these disorders is in the range of 0.5–1% per 150 individuals allowing them to be considered as widespread neurological diseases [10]. The reasons for such a rise are being actively discussed worldwide [11–21]. The study of ASD molecular mechanisms is a leading strategic goal of medicine aimed at early diagnosis of this nosological group.

The term “autism spectrum” was introduced by L. Wing in 80s of the XX century. It implied a continuum of the diseases accompanied by the disorders in communication, social interaction, social understanding, and imagination. Presently, the concept includes genetically and clinically heterogeneous psychic disorders united by the sign such as impaired social interaction. This is a group of the heterogeneous complex disorders of the nervous system development [22, 23].

The classification of the American Psychiatric Association (the Diagnostic and Statistical Manual of Mental Disorders, DSM-5, 2013) uses the term “spectrum” in the “Autism spectrum disorders” section (299.00 (F84.00) code) [24], since the disease manifestations strongly vary with the degree and age range ratio. In a new version of ICD-11, ASD is classified as a separate diagnostic unit. The essence of the disease has been debated up till now: whether it is a separate symptom, a negative syndrome, or an illness, a nosological form [25].

The main symptoms of autism are distinguished as follows [26, 27]:

decrease of communicative and social skills including speech communication;

stereotypy in behavior and speech; restricted, specific interests.

The described triad of symptoms can be accompanied by a weak visual contact, sensory dysfunction, impairment of cognitive and motor functions of various degree [28], self-aggression [29]. Concomitant disturbances of the gastrointestinal tract, sleep, and autoimmune pathologies are described [30]. Symptom intensity may vary widely.

As a rule, autism spectrum disorders are diagnosed at the age of 3–5 years, it is possible to reveal them at 1.5 years and in some cases at 6–12 months; the symptoms are preserved in adolescence and adulthood [31]. Currently, there are no highly sensitive and specific biological markers of ASD in medicine. The diagnosis is made on the basis of behavioral tests and medical history [32, 33], therefore the rate of the late diagnoses still remains high. Meanwhile, behavior correction is the most reasonable treatment, and the programs of early intervention seem to be more effective [34]. They can reduce maladaptivity of behavior and the severity of symptoms in childhood and improve the results of therapy in adult patients with ASD [35, 36].

The mechanism of ASD development is rather complicated, it includes, inter alia, heredity (64–91%) and genetic predisposition. More and more reports appear in the literature about new candidate genes [37, 38]. In recent years, an essential role of mitochondria and oxidative stress, as well as some amino acids, has been found in the autism pathogenesis. It is the last aspect that represents the least studied and rather a promising domain of the medical science. Great hopes in solving the problems of ASD diagnosis and treatment using the principles of evidence-based medicine are associated with the in-depth study of the neurochemical conception.

**The aim of the present review** was to systematize, summarize and describe in greater detail the main hypotheses of ASD development. The primary task was to reveal potential targets which could be used for the development of the novel methods for ASD diagnosis, prevention, and treatment.

## Systemic approaches to the study of autism spectrum disorders

The pathogenesis of autistic disorders is presently not clear enough. There exist a number of theories: genetic concepts, impairments of brain development associated with the impact of perinatal, neurochemical, immune, and other factors.

### Genetic hypotheses

For a long time, hereditary causes of autism have been considered dominating [39]. Despite the fact that genetic predisposition is determined only in 20% of cases [40], in 70–90% of cases a genetically determined character of the disease with the involvement of various genes is proposed [41–44]. Performing the meta-analysis of the NCBI databases, Chiurazzi et al. [45] have found out that not less than 2000 human genes are involved in ASD development and over 150 of them are located on X chromosome.

The risk of ASD development in siblings is known to be 20 times higher than in the general population. Twin studies have shown that family accumulation of autistic traits is a consequence of high heritability (h2:0.8). Patients were found to have changes in the number of chromosomes in separate cells, nucleotide substitutions in the structural genes, an abnormally changed gene copy number [46–49]. There was established the influence of parental age on the emergence of chromosome aberrations [50, 51].

The majority of ASD-associated genes determine functioning of the nervous system and the activity of certain proteins influencing the reproduction of genetic information [52, 53]. Mahfouz et al. [54] have shown in the network meta-analysis of the developing brain transcriptome that candidate genes determine functioning of the protein exchange and the development of mitochondrial dysfunction. At the same time, no statistical proofs showing the association of ASD with mutations in mitochondrial RNA have been found in the investigation of Álvarez-Iglesias et al. [40].

Studying the literature devoted to the genetic component of autism, two important facts were discovered.

Firstly, the effect of these mutations starts still in the process of the nervous system formation in the perinatal period and in early childhood as well. A number of mutations converge on the common path of the nervous system maturation in neurogenesis, axon development, and synapse formation [55] and these processes play a crucial role in the development of normal neuroplasticity [56].

Secondly, there appear first attempts to prove the existence of interconnection between genetic, ecological factors and epigenetics [57, 58]. According to Hannon et al. [59], hypoxia induces methylation of DNA at birth. Some mutations including rare ones are not called directly responsible for autism development but rather predisposing [60]. Several factors can activate their negative effect: fetus hypoxia, hemorrhage during pregnancy, complications during labor and delivery; parental age also matters a lot [61, 62]. Mother’s dietary habit, medicinal preparations taken in the antepartum period [63, 64], diabetes mellitus [65] and obesity [66], factors of unfavorable environment increase mutation rate [67]. Yasuda, Tsutsui [68], Skalny et al. [69, 70], Saldanha Tschinkel et al. [71] have shown in their works the link between ASD development and toxic effect of some metals. Some researchers [72, 73] studied the level of mercury in plasma, erythrocytes, brain, hair, and urine of patients with ASD and healthy people and have established that impaired processes of detoxication and metal excretion in the sick people resulted in its accumulation in tissues, stimulation of neuroinflammation, and disease development. In other investigations, abnormal concentration of chromium, magnesium, and zinc in hair and/or blood have been detected in patients with ASD compared to the control group [74], special importance was attached to zinc deficiency [75–77].

Colle et al. [78] have established a negative effect of pesticides (Paraquat and Maneb) on the expression of the main genes regulating the cell cycle. Similar results were obtained for insecticide Chlorpyrifos [79]. Bisphenol A activates the expression of 15 genes which are associated with ASD [80].

Gender (1:4, boys more frequently), metabolic and chromosomal diseases (Down syndrome, phenylketonuria, Rett syndrome) are referred to other factors.

Thus, according to the present concept, ASD develops under unfavorable exo- and endogenous factors in the critical periods of CNS ontogenesis when structural and functional qualitative changes providing the formation of more complex functions take place.

However, the mechanisms realizing the impact of these factors are not yet fully studied. Numerous genetic investigations and proposed diagnostic criteria are unfortunately not often reproducible and need further improvements.

### Disneuroontogenetic hypotheses

This direction in the study of ASD is related to the established disorders of the nervous system development at the early ontogenetic stages. Using current diagnostic methods (CT, MRI) and histological investigations of the nerve tissue samples, abnormalities of the brain development have been detected [81, 82]. It has been found that the brain volume and the volume of the temporal cortex in patients with ASD were reduced, there was a local expansion of the frontal cortex, enlargement of the volume of the lateral brain ventricles, impaired neuron maturation in the frontal cortex. Morphological changes of the cerebellum, hypoplasia of the cerebellar vermis and brainstem, callosum pathology, alterations of the periventricular white matter were also described.

Spread of diffuse changes in the brain results in disrupted interneuronal contacts, commissural and associative connections. For example, an alteration of functional coordination of cerebrocerebellar circuits in ASD in presence of the excitation/inhibition imbalance (glutamate + glutamine/glutamate + GABA ratio) has been shown [83, 84]. All changes mentioned above contribute to the genesis of the autistic syndromes [85–87].

The role of external factors provoking the CNS developmental disturbances not only at the genome level but also in the process of neuroontogenesis has been determined [88, 89]. These factors are numerous: infection or post-infectious condition of mother during pregnancy, birth injury, primary metabolic disorder, vaccinations, medicaments, industrial toxins, and others.

### Neurochemical hypotheses

Neurochemical hypotheses consider mainly ASD genesis from two sides: in connection with abnormal formation of neurotransmitter systems (mainly, glutamatergic) related to the deviations in the exchange of some amino acids and with the development of the oxidative stress [90].

Literature analysis has revealed the emergence of dysfunctions in mediator systems due to the exchange of amino acids and their derivatives (glutamate, glycine, GABA, serotonin, dopamine, noradrenaline) participating in the processes of synaptic plasticity [91].

#### The role of glutamate–glutamine–GABA exchange in autism spectrum disorders.

Glutamate (glutamic acid) is one of the main excitatory neurotransmitters responsible for learning, memory, behavior, motion, and sensation. Glutamate regulates induction of synapses and their interconnection with astrocytes, cell migration, synaptic spatial organization of the cerebellum, cell differentiation, and apoptosis.

Gamma-aminobutyric acid (GABA) is responsible for synaptic inhibition [92] being thus involved in brain development, cognitive activity, formation of attention. Consequently, inhibition of glutamate decarboxylase, GABA synthesis enzyme, leads to the rise of the glutamate amount in the nerve tissue while its excess results in overstimulation of glutamate receptors and, as a consequence, in neuronal excitotoxicity [93]. An increased content of glutamate and aspartate causing excitatory action has been found in autistic Kanner and Asperger syndromes. Glycine, which is a glutamate coagonist, facilitates excitotoxicity enhancement [94].

According to the data presented by Al-Otaish et al. [95], increase of GABA concentration by 80.65% (р=0.001) and the glutamate/glutamine ratio by 56.98% (р=0.027) was noted in the blood plasma of the ASD patients compared to the control. Glutamine is a precursor of glutamic acid and GABA, while glutamate amination serves as a way of protection against excitotoxicity promoting normal brain development. Increase of the glutamic acid concentration is likely to occur due to the failure of its re-uptake by astrocytes.

Not only glutamate concentration plays an important role but also a prolonged imbalance in the excitatory and inhibitory mechanisms of GABA and glutamate [96, 97]. GABA is involved in the pathophysiology of autism spectrum disorders. Extremely high GABA levels have been shown in plasma and a concurrent reduction of its level in the brain, besides, there has been detected a decrease of GABA receptor activity in hippocampus, neocortex, and cerebellum, appearance of GABA receptor subunits in the blood [98]. A reverse situation is observed when glutamate decarboxylase enzyme is inhibited. It has been established on the animal model [96], that inhibition of the GABAergic system leads to the enhancement of the neuronal activation (aberrant activation) causing cognitive capability impairment. In another experiment on the model animals [29], reduction of GABA concentration in the blood was shown.

The ratios of the listed metabolites in the blood are proposed as ASD biochemical markers [99].

***Serotonin*** (tryptophan derivative) in the nervous system is responsible for cell proliferation and differentiation; migration; apoptosis; synaptogenesis; maturation of the prefrontal cortex, parts of the limbic system, corpus callosum; regulation of emotions, mood, memory, and learning. Muller et al. [99] have noted the decrease of serotonin level in the model animals by 22.48% relative to the control, and also a rise in platelet-bound serotonin. Its high level in the blood (hyperserotonimia) was proposed by the authors as a biologic ASD marker. They have also demonstrated the interconnection between the serotonin level and intensity of symptoms such as stereotypy in behavior and speech, extremely restricted interests. Application of this biogenic amine is proposed as a target for targeted pharmacotherapy.

Too high efficacy of the serotonin reuptake transporter (SERT) in autism was also reported [100, 101], this is a genetically inherited abnormality.

***Catecholamines*** (tyrosine derivative) in the nervous system are neurotrophic factors, neurotransmitters, modulators of the neural and humoral regulation. Dopamine, in particular, is responsible for attention, emotional reward, sensory and motor functions, communication skills which are impaired in autism [102]. The risk of autism development is supposed to increase in case of entire dopamine homeostasis disturbance due to the imbalance of dopamine receptor distribution in the basal ganglia and prefrontal cortex — parts of the brain implicated in the cognitive function [103]. Thus, mutation in the protein gene, a dopamine reuptake transporter, is one of the causes of autism development. Another reason may consist in a low activity of dopamine-β-hydroxylase, catalyzing the conversion of dopamine to noradrenaline. The enzyme is controlled by a single gene *DβH*. A low activity of this gene in the perinatal period creates the conditions for autism development [98]. It is connected with the fact that noradrenaline system influences attention, memorizing, mobilization of the intellectual and emotional activity, motivated behavior, imaginative thinking [104].

#### The role of other amino acids, neurotransmitters, and hormones in the pathogenesis of autism spectrum disorders.

Obviousness of implication of some amino acids and biogenic amines in ASD development should be recognized. Taurine (inhibiting neurotransmitter), arginine (nitric oxide donor), methionine (choline formation) have also been noted in maintaining the balance between the inhibitory and excitatory systems regulating neuroplasticity [95].

Stroganova et al. [105] suppose that cholinergic transmission insufficiency may be the cause of attention impairment in ASD people throughout their life since the cholinergic system plays an important role in realization of this function. The authors think that the deficit of nACh receptors may be caused by the reduction of gene expression but posttranscriptional causes may also play a role. Besides, they suppose that abnormalities in neurexin-1 result in incorrect positioning of the nACh receptors in postsynaptic membrane.

Having obtained the results on an ASD animal model Wang et al. [106] proposed to use cholinergic receptor agonist as a modulator of the deficit of social and repetitive behavior.

Oxytocin and vasopressin, hormones secreted in hypothalamus and stored in the posterior lobe of hypophysis, are considered among the factors participating in ASD development. The interest to them was not accidental. Oxytocin is an important modulator of human associative behavior including social skills, pair bonding, parental affection and friendship, confidence formation [107]. Oxytocin is involved in the regulation of repetitive and affiliative behavior which is a key factor for autism development. The work [108] demonstrates decrease of the oxytocin level in the blood and this correlates with methylation in the promotor area of the oxytocin receptor gene (*OXTR*) in individuals with ASD diagnosis. This epigenetic modification may contribute to the formation of autistic social and behavioral phenotypes.

Dysfunction of dopamine-oxytocin systems is supposed to lead to the lack of striving for rewards in patients with ASD, therefore the reward system during treatment may be ineffective [109].

Carson et al. [110] suggest using the level of another hormone, vasopressin, in the blood as a biomarker of social interaction impairment in ASD, and vasopressin signaling disruption, especially in men, as a risk factor of ASD development. Rutigliano et al. [111] came to the conclusion that vasopressin preparations may be promising for communicability improvement, however, they noted inconsistency of data in various studies about the role of vasopressin in ASD development.

#### Glutamate excitotoxicity.

Describing neurochemical mechanisms of ASD development, glutamate excitotoxicity should be distinguished as one of the prime causes [83].

Excessive amount of glutamate, aspartate, and other excitatory neurotransmitters leads to overexcitation of ionotropic glutamatergia due to NMDA (N-methyl-D-aspartate) receptors and AMPA (α-amino-3-hydroxy-5-methyl-4-isoxazolepropionic acid) resulting in the increase of calcium concentration in cytosol. Calcium ions activate the inducible isoform of NO synthase, increasing nitric oxide concentration which, in its turn, activates phosphorylation of protein kinase C and phospholipase A2.

A succession of these events leads to the enhancement of lipid peroxidation (LPO). Free radicals may inhibit oxidative phosphorylation decreasing ATP production in mitochondria and triggering apoptosis cascade. The ATP products such as 4-hydroxynonenal (4-HNE) may interact with synaptic proteins and disturb transport of glucose and glutamate, increasing sensitivity of the nerve cells to excitotoxicity [112].

The imbalance of excitation and inhibition at the cell level causes the appearance of new properties in the neuronal network. Levin, Nelson [97] believe that these properties take into consideration a more complete genetic and cellular background than the results at the level of separate genes or cells, and are capable to be diagnostic biomarkers.

It is notable that the child’s brain is more vulnerable to excitotoxic effects as the developing nervous system contains more synaptic receptors of glutamate than at birth and their quantity decreases with age [93]. Therefore, glutamate excitotoxicity plays an important role in ASD development, in the opinion of numerous researchers.

### Mitochondrial dysfunction and oxidative stress

The vitality of the hypothesis is confirmed by the fact that mitochondrial dysfunction and oxidative stress, as the links of pathogenesis, may influence social and cognitive impairments in autism [113, 114]. Patients with ASD have been found to have decreased activity of enzyme complexes of respiratory chain and other enzymes of energy exchange: creatine phosphokinase, pyruvate dehydrogenase [115]. This is associated with low expression in the brain, especially in the cerebellum, frontal and temporal cortex. As a consequence of mitochondrial dysfunction, the levels of lactate [116], pyruvate, alanine, and ammonia rise. El-Ansary et al. [30] proposed to use ratios of various indices of exchange in mitochondria as additional markers of ASD-associated dysfunction. According to the study of these researchers, the ratios of the indices characterizing the efficiency of the respiratory chain, the activity of apoptotic and antioxidative processes such as NADH dehydrogenase/caspase-7, GSH/GST, and NADH dehydrogenase/coenzyme Q10 were most evident in patients with ASD compared to the control group.

At the same time, abnormally high concentrations of lipid peroxides were determined in these areas of the brain being evidence of oxidative stress induction [112].

Patients with ASD and animal models were noted to have increased number of reactive oxygen species (ROS), LPO activation, and accumulation of its products [117]. At the same time, the activity of antioxidant systems (glutathione, superoxide dismutase, and glutathione peroxidase, coenzyme Q [118]) is reduced. Positive correlation has been shown between the increase of ROS and reduction of antioxidative antioxidant activity and autism severity which is likely to be due to the activation of apoptosis and aberrant synaptogenesis [119]. The activation of free-radical processes alters the activity not only of the energy metabolism enzymes. Thus, the reduction of Na^+^/ K^+^-ATPase activity increases the glutamate receptor sensitivity which, in its turn, leads to depolarization and increased excitation of the nervous system in the regions connected with motor activity and promotes calcium output. The enumerated processes are part of glutamate excitotoxicity, thus neuroinflammation, oxidative stress, and glutamate excitotoxicity are part of the signaling system associated with ASD. Hypoperfusion, the extent of which correlates with autism severity, is also considered to be a cause of this dysfunction. Chauhan et al. [112] have supposed that hypoperfusion is caused by vascular inflammation.

To correct mitochondrial dysfunction and oxidative stress, various types of supplements to the diet including antioxidants (coenzyme Q, 5-aminolevulinic acid) are proposed by some authors [119, 120]. Choline [7] is recommended for cholinergic system activation, adequate prenatal and postnatal supply of different polyunsaturated fatty acids (especially docosahexaenoic and ω-3) for normal brain growth and development. Vitamin B_12_ usually accompanies folate deficit causing neurological disorders and congenital defects [108]. В_12_ deficit is in inverse proportion to the homocysteine level which is known to be a modulator of the lipid metabolism [91]. Increased vulnerability to the oxidative stress may impair vitamin D metabolism and other cholesterol derivatives [121]. In one of the studies [122], the role of mutation of vitamin D receptor gene is investigated. The deficit of this vitamin leads to the enlargement of the size and changes in the shape of the brain and also increases the probability of developing autoimmune processes. Patients with ASD are often diagnosed with a reduced level of vitamin D_3_ in childhood therefore its additional intake is also recommended as a method of treatment and prevention of ASD [123].

Melatonin (tryptophan derivative) is functioning as an electron donor reducing electrophilic radicals. There is experimental evidence that it acts as an indirect antioxidant stimulating antioxidant enzymes. Several studies have shown that melatonin diminishes chronic and acute inflammation, NO amount, and the level of malonic dialdehyde [124].

Thus, the oxidative stress and mitochondrial dysfunction occupy an intermediate position between several factors of external environment, glutamate excitotoxicity, and neuroinflammation in pathochemistry of ASD.

### Immune hypothesis

Immune hypothesis suggests [125–127] the probability of autoimmune reaction (high level of antibodies to neuroantigens) promoting disturbances in the nervous system development. On the other hand, a high titer of autoantibodies is a consequence of natural immunity activation. The severity of patient’s condition correlates with the rise of acute-phase protein level, ASD markers. Besides, the decrease of the number and activity of NK-cells contributes to the reduction of resistance of children with autism spectrum disorders to viral infections, especially, neurotropic, which may be dangerous for the nervous system development in certain critical periods. It is proved by the presence of brain-specific autoimmune antibodies in some ASD children, and also by the increased rate of autoimmune diseases in the families with autism [128].

Additional investigations [129] demonstrated the reduction of the Purkinje cell number due to microglia activation and further neuroinflammation causing oxidative stress because of the emerging disbalance of glutamatergic and GABAergic transmission ratio as well as the development of excitotoxicity. At the same time, increase of concentration of inflammatory cytokines such as tumor necrosis factor α (TNF-α) in the blood, brain, spinal fluid has been noted in the works [129, 130]. It is one of the cytokines synthesized by the interaction between the cells of the immune system and CNS. The increased levels of TNF-α in the serum are also correlated with the serum levels of adipokines such as visfatin and resistin. Increase of IFN-γ level, interleukins (IL-6, -8 and -12 [103]; IL-17, -1, -1β, -5, -8, -12, -13, -23 [131, 132]) has been also registered in people with autism spectrum disorders. Of special importance in the formation of antibodies is IL-17А associated with the induction of neutrophilic recruiting chemokine synthesis. Additionally, osteopontin which was detected in children with autism induces Th17 synthesis by the cells of myelin antigen-specific IL-17 via specific osteopontin receptors (integrin-β3 receptors) on T cells [131, 132].

IL-6 is a neuropoietic cytokine influencing neuronal proliferation, synapse formation, differentiation, and migration. It is supposed that activation of the immune system which may be connected with ASD development in children depends on mother’s IL-6. Besides, IL-6 plays a critical role in the development and modulation of autistic behavior due to the disorders in neuronal network formation [107].

The recent study [130] has discovered the role of gestational maternal infection and congenital immune responses to the infection in the pathogenesis at least of several ASD cases.

The investigation carried out by Bryn et al. [8] has shown that a cytokine profile in patients with ASD does not differ from that in the control group. However, the authors note the differences in ASD subgroups (IL-8 and IL-10) illustrating additionally the heterogeneity of this group of pathologies. The multiplex cytokine analysis is also proposed to be used as an ASD biomarker [132].

In recent years, disruptions of endocannabinoid signaling pathway in the formation of neuroinflammation have been explored [133].

The growing interest in the role of chronic inflammation in neurological disorders is connected to the fact that generalized alterations in oxidative/antioxidant status, as well as immune, endocrine systems, and neuromediator transmission in the brain take place in inflammation [134]. Immune system disorders may become a new target in autism treatment [135].

### Opioid hypothesis

The emergence of this theory which got the name “exorphine hypothesis of autism” is linked to the discovery of casomorphins (products of milk casein hydrolysis), gliado- and glutenomorphins (products of grain crop protein hydrolysis) which are dietary-derived opioid peptides. Data on the interconnection of autism with the impairment of the intestine barrier permeability and the decrease in the activity of proteolytic enzymes breaking down proteins and peptides underlie the hypothesis.

These disorders result in an increased level of the non-spliced peptides, caso-gliado- and glutenomorphins, in the blood. The generated exorphins penetrate the brain with the blood current where they affect the opioid and related neurochemical systems resulting in the development of autism spectrum symptoms [136, 137]. Gluten and casein intolerance is supposed to act as a triggering mechanism of inflammation and to promote ASD development [138].

### Hypothesis on local translation impairment, the role of the mTOR signaling pathway

Synaptic plasticity is considered to be implemented by protein biosynthesis changes. Thus, Phelan–McDermid syndrome is associated with the loss of functionality by gene *Shank3*. Some ASD may be connected with the dysfunction of neuroligins and neurexins. Angelman syndrome (happy puppet syndrome) is associated with the excess of target-proteins, Arc in particular, in the postsynaptic space. Its function is the internalization of AMPA-subtype glutamate receptors [102].

There is an opinion about the role of microRNA in the formation of aberrant neuroplasticity since microRNA is capable of inhibiting protein synthesis by transcription repression and destabilization and destruction of mRNA. The necessity of continuing investigations in this direction is also noted by the authors [139].

A local translation is a finely regulated process; the central role in the regulation initiation is played by mTOR (mammalian/mechanistic target of rapamycin) signaling pathway which integrates numerous intracellular and extracellular signals including growth factors, nutrients, stress, infections and is also implicated in the formation of memory and long-term synaptic plasticity. The disruption of separate links of this pathway leads to the development of autism spectrum disorders [140, 141]. Impairment of mTOR inhibition may also result in ASD: for example, pathways PTEN (phosphatase and tensin homolog deleted on chromosome 10), since PTEN (lipid phosphatase), a negative regulator of the mTOR signaling pathway, is involved in neuroplasticity [142].

**Figure 1:**
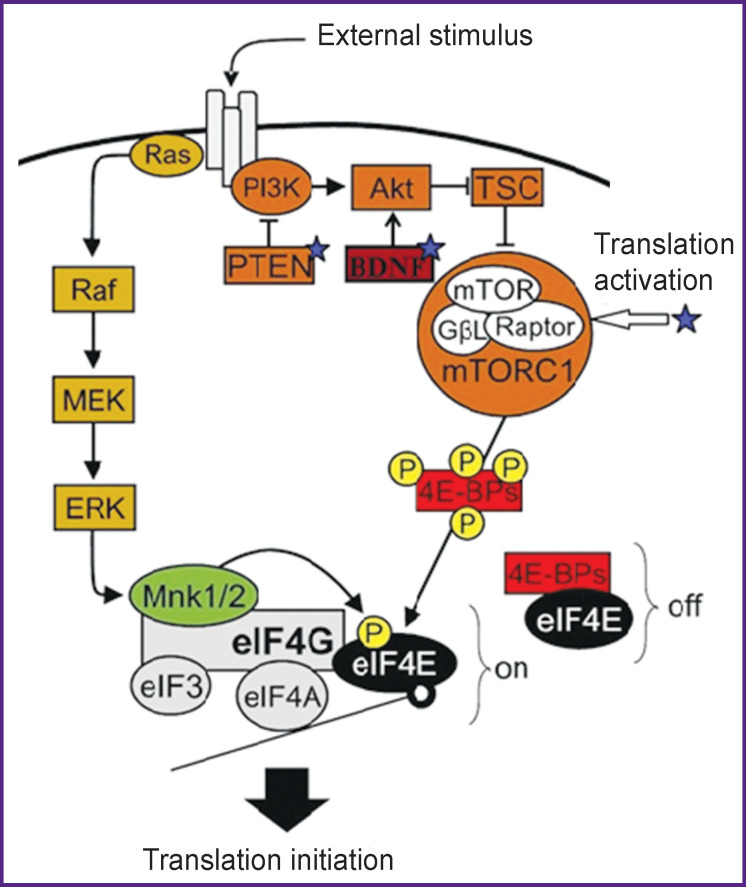
The role of mTOR pathway in regulation of translation and factors influencing it (according to Amorim et al. [141] in the authors’ modification) Ras — small GTPases; Raf — proto-oncogenic serine/threonine-protein kinase; BDNF — brain-derived neurotrophic factor; 4E-BPs — eIF4E-binding proteins; Akt — protein kinase B (PKB); eIF3 — eukaryotic initiation factor 3; eIf4A — eukaryotic initiation factor 4A; eIf4E — eukaryotic initiation factor 4E; eIF4G — eukaryotic initiation factor 4G; ERK — extracellular signal-regulated kinase also known as mitogen-activated protein kinase (MAPK); GβL — G protein β subunit-like; MEK — mitogen-activated protein kinase kinase; Mnk1/2 — mitogen-activated protein (MAP) kinase-interacting serine/threonine-protein kinases 1/2; mTOR — mammalian target of rapamycin; mTORC1— mammalian target of rapamycin complex 1; off — repression of translation; on — active translation; P — phosphorylation site; PI3K — phosphoinositide 3-kinase; PTEN — phosphatase, product of *PTEN* gene; RAPTOR — regulatory-associated protein of mTOR; TSC — tuberous sclerosis protein; asterisks show the sites of possible impairments contributing to ASD development due to mTOR overactivation

Fragile X syndrome is linked to mTOR hyperactivation. After the application of mTOR inhibitors (everolimus and sirolimus, rapamycin) on the models of the mutant mice with increased mTOR activation, an improved condition of the animals was observed [143]. Notably, even in adult model mice, treatment with rapamycin led to prolonged regeneration of potentiation; improvement in behavior and learning also was noted illustrating the reversibility of behavioral disorders in autism [144] (Figure 1).

It is interesting to note that low levels of mTOR activity are associated with Rett syndrome [140]. BDNF protein (brain-derived neurotrophic factor) binds to the В (TrkB) tyrosine kinase receptor and activates a variety of intracellular signaling pathways including mTOR. The reduction of the protein level and mRNA BDNF is considered to contribute essentially to the Rett syndrome pathophysiology. This protein is being studied in other ASD [145].

Armeanu et al. [146] report that BDNF participates actively in neuroplasticity and normal development of the nervous system. However, multiple previous investigations of its concentration in the blood plasma and serum show contradicting results in the ASD individuals. The meta-analysis of the available data demonstrates an increased BDNF level in the blood of patients with ASD in contrast to schizophrenia or bipolar disorder. The authors explain the obtained data by the activation of the protein synthesis in synapses in autism.

## Conclusion

The conducted analysis of the current literature sources on the mechanisms of autism spectrum disorders proves polyetiologicity, complexity, and multifactorial nature of this group of diseases related to the disorder of brain development and functions. The majority of researchers today are convinced that autism is a genetically determined illness. However, not every individual possessing candidate genes develops autism spectrum disorder, and if the disease develops it may have different degree of severity. The epigenetic factors and factors of external environment are able to activate by various ways the conversion of the nervous system to the autistic type. These factors must act at the early stages of the nervous system formation — at the perinatal and early postnatal periods, i.e. actually we may speak about aberrant neuroplasticity in the critical moments of the nervous system development. However, the mechanisms by which these factors influence the development of aberrant neuroplasticity in autism spectrum disorders need further studies and clarification.

**Figure 2:**
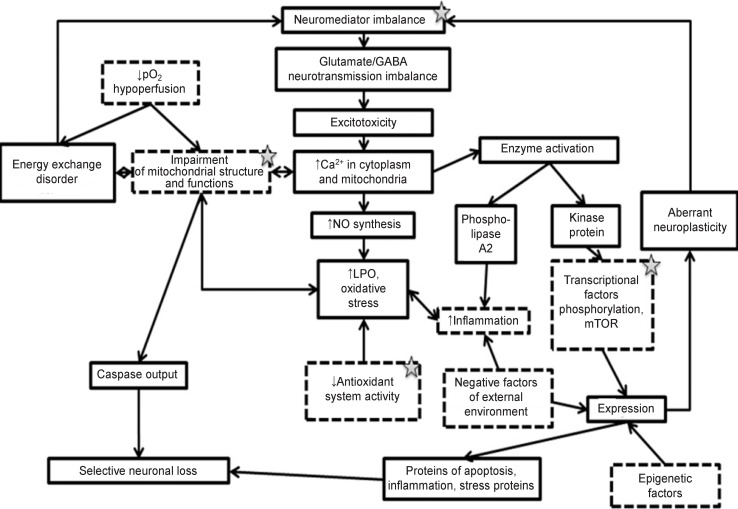
Involvement of glutamate excitotoxicity in various ASD-forming mechanisms Asterisks note the factors which may be activated due to genetic causes; dotted lines designate the supposed initial causes of ASD

In 2013, Essa et al. [93] showed that excitotoxicity and oxidative stress are pathological events which modulate interconnection between genetic, ecological, and immunological risk factors for autism. A large number of the explored mechanisms of ASD pathogenesis are in the bulk linked to excitotoxicity and impairment of the cell redox balance with activation of the oxidative stress reactions. But the question whether the cause inducing excitotoxicity and oxidative stress (for example, neuroinflammation) is primary or vice versa the oxidative stress and excitotoxicity provide conditions for the development of ASD is solved by the researchers in different ways. A single picture on this problem has not been formed yet and undoubtedly requires special attention to this issue. It is worth mentioning that the mechanism of excitotoxicity development is an integral part of the molecular mechanism of aberrant plasticity (Figure 2).

In this connection, the possibility of using glutamate, GABA, oxytocin, serotonin, dopamine; combination of IL-6 and serotonin, ratios of various interleukins; enzyme activity of mitochondrial electron transport chain and, so on, is considered as diagnostic criteria, meaning that there is no unified biosensor at present time which would be highly selective and sensitive in autism spectrum disorders. This is extremely important as medical rehabilitation must start at the very early stages of autistic disorder formation. Additional investigations, meta-analysis of data using specialized databases (“dry biochemistry”) are required to form a complete picture of the ASD nature. Their high prevalence demands stirring up researcher activity in this direction. This will help elaborate a more effective therapeutic strategy in regeneration of physiological homeostasis in patients as well as in creation of unified generally recognized diagnostic criteria within the frames of evidence-based medicine.
